# Landing wise program: feasibility study protocol for Parkinson’s disease

**DOI:** 10.3389/fmed.2023.1247660

**Published:** 2023-10-17

**Authors:** Júlio Belo Fernandes, Josefa Domingos, Carlos Família, Cátia Santos, Diana Santana, Francisco Gregório, Inês Costa, Joana Afonso, Lúcia Matos, Solange Marques, Tânia Santos, Sónia Fernandes, Isabel Santos, Natacha Sousa, Catarina Ramos, Catarina Godinho

**Affiliations:** ^1^Egas Moniz Center for Interdisciplinary Research (CiiEM), Egas Moniz School of Health & Science, Almada, Portugal; ^2^Nurs* Lab, Almada, Portugal; ^3^Molecular Pathology and Forensic Biochemistry Laboratory, Caparica, Portugal; ^4^Department of Nursing, Centro Hospitalar de Setúbal, Setúbal, Portugal; ^5^Department of Nursing, Hospital Garcia de Orta EPE (HGO), Almada, Portugal; ^6^Department of NursingClínica Cuf Almada (CUF), Caparica, Portugal; ^7^Careceiver and NOVA Medical School (MS) & NOVA School of Business and Economics (SBE), Lisbon, Portugal; ^8^ARS LVT, ACES Arrábida, UCC Palmela, Palmela, Portugal; ^9^ARS LVT, ACES Lisboa Norte, UCC Integrar na Saúde - ECCI Benfica, Lisbon, Portugal; ^10^LabPSI, Almada, Portugal

**Keywords:** Parkinson’s disease, accidental falls, accident prevention, exercise movement techniques, cognitive behavioral therapy, safe landing, rehabilitation, physiotherapy

## Abstract

Regardless of the benefits of fall prevention programs, people with Parkinson’s disease (PD) will still fall. Therefore, it is crucial to explore novel therapeutic approaches that are well-accepted and effective for addressing fall risk and the fear of falls among this population. The present study aims to assess the feasibility of the Landing Wise program as a therapeutic intervention for reducing the fear of falling in people with PD. A mixed-methods study will be conducted using convenience sampling to recruit 20 people with PD with a moderate concern of falling from a Parkinson’s Patients Association. In addition to usual care, participants will attend 2 days per week, 90 min group sessions for 8 weeks. The intervention combines group cognitive behavioral intervention with the training of safe landing strategies. Feasibility will be assessed by six key domains (recruitment strategy and rates, enrollment, retention, acceptability, reasons for decline/withdrawal, and adverse events). Quantitative data will be analyzed using descriptive statistics to characterize the sample, followed by inferential statistics to evaluate differences in the Short Falls Efficacy Scale-International Scale, Movement Disorder Society Unified Parkinson’s Disease Rating Scale, Timed Up Go, 6-Minutes Walking Distance, and fall frequency and severity scores between baseline and final assessment. Qualitative data will be analyzed using an inductive thematic analysis process. There is a growing interest in developing new effective therapeutic approaches for people with PD. If proven program feasibility, this study precedes a randomized controlled trial to establish the effectiveness of the Landing Wise program.

## 1. Introduction

Parkinson’s disease (PD) is a chronic degenerative disorder of the central nervous system that primarily affects the motor system, triggering involuntary or uncontrollable movements, like tremors, rigidity, bradykinesia, and difficulty with balance and coordination (1, 2). However, PD can also cause non-motor complications such as cognitive impairment, sleep disorders, mental health disorders, pain, and sensory disturbances (2). In the last two decades, PD prevalence has doubled, and it is estimated that in 2019 affected over 8.5 million individuals (1, 3). Progression of PD symptoms leads to restrictions in many life areas, ultimately resulting in high rates of disability and care requirements (4).

In 2019, the WHO (1) estimated that PD resulted in 329,000 deaths, showing an increase of over 100% in the last two decades, and was responsible for 5.8 million disability-adjusted life years, resulting in a rise of 81% over the same period, escalating faster than for any other neurological disorder worldwide.

Falls are common among people with PD (5, 6), and, notably, the fall rate in this population is often higher than that observed in older adults without PD (5, 7). Consequently, they can experience fearful anticipation of falls, developing a fear of falling (FOF) (8, 9). The FOF can ultimately reduce balance performance and limit the person’s activity levels (8, 10), compromising one’s quality of life (11). Fall prevention programs mainly targeted intrinsic (e.g., muscle weakness, balance problem) or extrinsic (e.g., environmental hazards) fall risk factors (9, 12). Despite the benefits of these programs, it is crucial to highlight that participants within these programs are still at risk of falling (13).

It is vital to develop novel therapeutic approaches that are technically feasible, economically valuable, and culturally, ethically, and socially accepted for addressing fall risk and the FOF among this population (14–16).

Recent studies show that it is possible to reduce the fear of falling among older adults (17, 18). In addition, there is evidence that cognitive behavioral therapy (CBT) can effectively reduce the fear of falling, with significant immediate retention effects for up to 12 months (19–21). CBT is a psychotherapeutic skills-based, non-pharmacological treatment aimed at modifying individuals’ thoughts and behavior by teaching practical strategies to support the individual more effectively in navigating daily challenges (22). In the Landing Wise program context, CBT principles and techniques are seamlessly integrated to address the physical aspects, such as safe landing strategies, and the psychological aspects, including FOF and anxiety, commonly experienced by individuals with PD.

The Landing Wise program takes a comprehensive approach that goes beyond physical training. It delves into the cognitive and emotional dimensions of FOF and anxiety, helping participants explore the intricate connections between their thoughts, feelings, and behaviors. Doing so empowers them with practical strategies to navigate daily challenges more effectively and adjust thought patterns—particularly those marked by negativity or behaviors like social isolation and withdrawal (23).

Previous studies have shown that CBT is a feasible treatment for anxiety and depressive symptoms in people with PD (24–27). More importantly, when applied to address FOF, CBT may yield broader positive outcomes, potentially reducing falls and enhancing overall activities of daily living (28, 29). The versatility of CBT allows for deploying a wide range of strategies can be used in CBT, such as cognitive restructuring, training of coping skills, or practicing new skills that can be used in real-world situations to help people with PD to overcome these patterns. For instance, as part of the Landing Wise program’s integration of CBT, individuals grappling with FOF may actively practice safe landing strategies. Not only do these strategies provide tools for safe fall management, but they also reduce the impact load of a fall. A systematic review by Moon and Sosnoff (13) synthesized findings from 13 studies on safe landing strategies, demonstrating their potential to reduce the risk of injury during falls significantly. The authors concluded that landing strategies significantly decrease the impact load during a fall and might effectively reduce the impact load of falling.

Considering that previous studies have shown the positive effects of CBT and practice safe landing strategies on people at risk of falling, here, we combine the two approaches in a program. Therefore, in this study, our primary aim is to assess the feasibility of the Landing Wise program as a therapeutic intervention for reducing FOF in people with PD. Secondary objectives include assessing the program’s preliminary effects on the people with PD.

## 2. Materials and methods

### 2.1. Study design

This study is a mixed methods study using quantitative and qualitative assessments. To ensure the quality of the research protocol report, we will use The Good Reporting of A Mixed Methods Study (GRAMMS) checklist (30).

### 2.2. Study setting

The intervention will be delivered in a gymnasium of a Day Care Unit from a Private Institution of Social Solidarity in the region of Lisbon and Tagus Valey in Portugal that caters to a population of over 80,000 people.

### 2.3. Sampling and recruitment

The study population consists of people with PD recruited from the Parkinson Patients Association and the outpatient neurology units from two hospital centers in Lisbon and Tagus Valley. The sampling method selection will be non-probabilistic by convenience. All eligible candidates will be invited to join the program.

Participants will be included if they fulfill the inclusion criteria:

- Diagnosis of idiopathic PD (Movement Disorder Society PD criteria) (31);
- Hoehn and Yahr stages II–IV (32);
- Age above 18;
- Moderate concern of falling with the Short Falls Efficacy Scale International (Short FES-I) >9 (33);
- A Montreal Cognitive Assessment (MoCA) score > 25 (Normal cognition) (34);
- Able to tolerate a minimum of 45 min of exercise (Following recommendations form PD guidelines) (35);
- Able to communicate with the investigator, to understand and comply with the study procedures;
- Willing and able to provide written informed consent to participate and understand the right to withdraw their consent at any time without prejudice to future medical care.

Participants will be excluded if they have any of the following:

- A MDS-UDPRS Part III item 3.12 score > 3 (Severe postural instability: very unstable, tends to lose balance spontaneously or with just a gentle pull on the shoulders);
- Severe cognitive difficulties and significant active psychiatric problems that aggravate when exercising;
- Severe hearing or visual impairment;
- Missed two consecutive sessions.

Our study aims to recruit 20 participants in total. This sample size was calculated using G*Power (36), taking into account a large effect size (dz = 0.8), an alpha level (α) of 0.05, and a statistical power (1-β) of 0.8. These calculations were performed for both the two-tailed matched pairs t-test and the two-tailed matched pairs Wilcoxon signed-rank test, which will be employed in the quantitative data analysis. Further details about these analyses are provided in the data analysis section below. Both tests indicated a required sample size of 15 participants. To account for a possible dropout rate of 25%, we increased the sample size to 20 participants.

**Figure 1:**
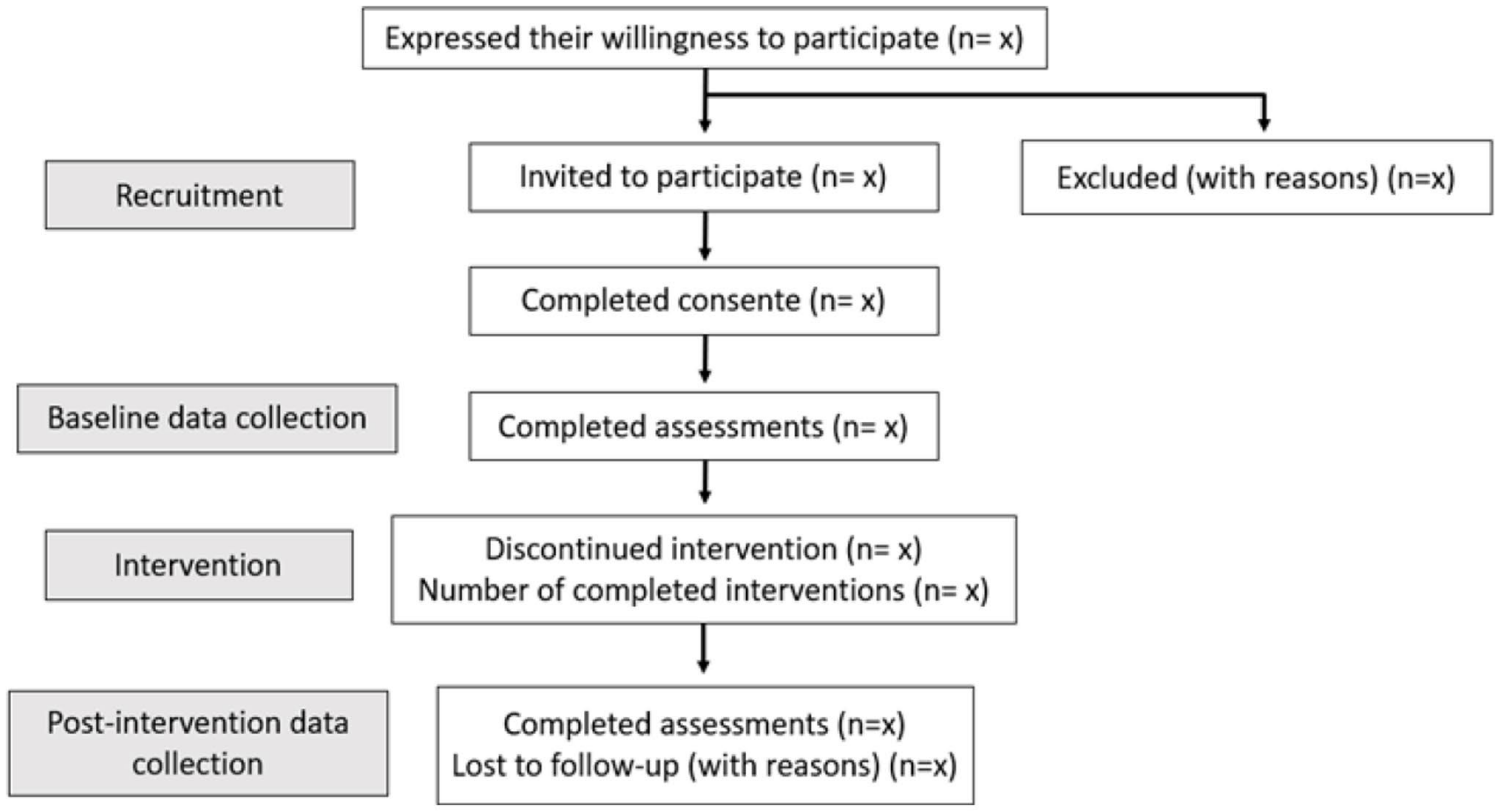
Flow diagram.

In the first stage of recruitment, healthcare professionals (nurses, physiotherapists, and physicians) will be responsible for screening and identifying suitable participants based on the data in the patient’s clinical file (diagnosis, staging of the functional disability associated with PD, and clinical data). These professionals will introduce the study to potential participants and provide an information sheet containing the study aims and procedures. A research team member will contact potential participants who have applied for the recruitment process via telephone to present comprehensive information regarding the study procedures and verify their willingness to participate. We recommend taking a minimum of 24 h to consider the advantages and disadvantages of participating in the study and formulate questions before deciding on participating. Once all questions have been answered, potential participants will be asked to sign an informed consent. Access to patient clinical data will only be granted to the researcher at this stage. Figure 1 shows a flow diagram for participants.

### 2.4. Intervention

The intervention consists of an 8-week program with 90-min group sessions held twice a week. The program combines group CBT with training in safe landing strategies, thus ensuring each component receives dedicated 45-min sessions (35, 37, 38). This intervention will supplement the usual care.

The intervention will be delivered by a rehabilitation nurse with PhDs in Nursing Sciences and Psychology, trained in Cognitive behavioral therapy, and experienced in motor and cognitive exercise programs. Following European Parkinson’s guidelines (35), we will consider eight people with PD as the optimum number of participants per group.

Assistance to the instructor will be provided by two nurses that are receiving their master’s training in rehabilitation nursing. In addition, the students will receive 8 h of CBT training conducted by a cognitive-behavioral therapist.

To keep the program challenging and engaging, the instructor will gradually raise the complexity of the exercise session accordingly to the participants’ willingness and improved condition. The instructor will use the Borg Effort Perception Scale to assess the participants’ perceived effort through sessions (39). Participants can achieve an intensity rating between 14 (Somewhat Hard) and 17 (Very Hard) on a 20-point Borg scale, indicating a range from somewhat challenging to very hard perceived exertion during exercise (40).

The CBT will be based on the FOF management model (41) that illustrates how community-dwelling older adults think, feel, and act when facing FOF. According to this model, the FOF arises when the person believes falling is an inevitable part of aging. These misconceptions may lead older adults to withdraw from social activities instead of focusing on strategies to manage the problem. The FOF model indicates that older adults may embrace different strategies: display psychosomatic symptoms, adopt an attitude of risk prevention, pay attention to environmental safety, and modify their own’s behavior. The FOF will be relieved when the person is satisfied with the outcomes (41).

The program syllabus will be designed based on prior research (42, 43), aiming to restructure misconceptions to foster a positive view of fall risk management, increase self-confidence and physical wellness concerning falling, and a sense of control over falling. The sessions will cover the following themes: (1) introduction; (2) associations with falls and fear of falling; (3) participant’s view of FOF; (4) cognitive restructuring; (5) strategies to manage fall risk; (6) strategies to manage FOF; (7) application of strategies in daily life; and (8) problem-solving (learning how to fall, stand up and call for help).

Each session will be carefully planned by a team composed of a cognitive-behavioral therapist, a rehabilitation nurse, a physiotherapist, and an exercise physiologist, experts in PD, who will develop a guide to help manage each session. Then, a panel of experts in CBT will assess the guide’s appropriateness and contents. The structure of the CBT sessions is described in Table 1.

**Table 1:** Structure of the CBT sessions.

Phase 1: Warm-Up (5 min)	Social interaction (e.g., greeting friends). Setting a positive and supportive tone for the session. Introduction to the session’s topic. Brief review of the previous session (if applicable). Engaging in a warm-up activity to promote relaxation and focus.
Phase 2: Cognitive restructuring approach (20 min)	Explanation of the concept being addressed. Discussion, group exercises, or role-playing related to the skill. Identifying and addressing cognitive and emotional aspects (e.g., fear of falling, negative thought patterns).
Phase 3: Sharing and Support (15 min)	Encouraging participants to share their experiences or insights related to the session’s topic. Providing support and feedback within the group setting.
Phase 4: Closing (5 min)	Summary of the key points covered in the session. Homework assignments or practice exercises for participants to work on until the next session. Ending the session on a positive and motivating note.

After completing the CBT, the initial stage of safe landing training commences with a 10-min physical warm-up session aimed at reducing the risk of injury. This is followed by a 10-min improvisation phase, wherein participants will have the freedom to move in accordance with specific instructions (such as bilateral movements, large and big movements, movements that are not usually carried out in everyday life activities, as getting up and down from the floor, etc.).

All participants will be equipped with protective gear for the second stage, including a wrist guard and neck protector. The intervention methods consist of 20 min of safe landing training exercises aiming to teach participants how to fall in such a manner to alleviate the impact severity and minimize the risk of injury.

**Figure 2:**
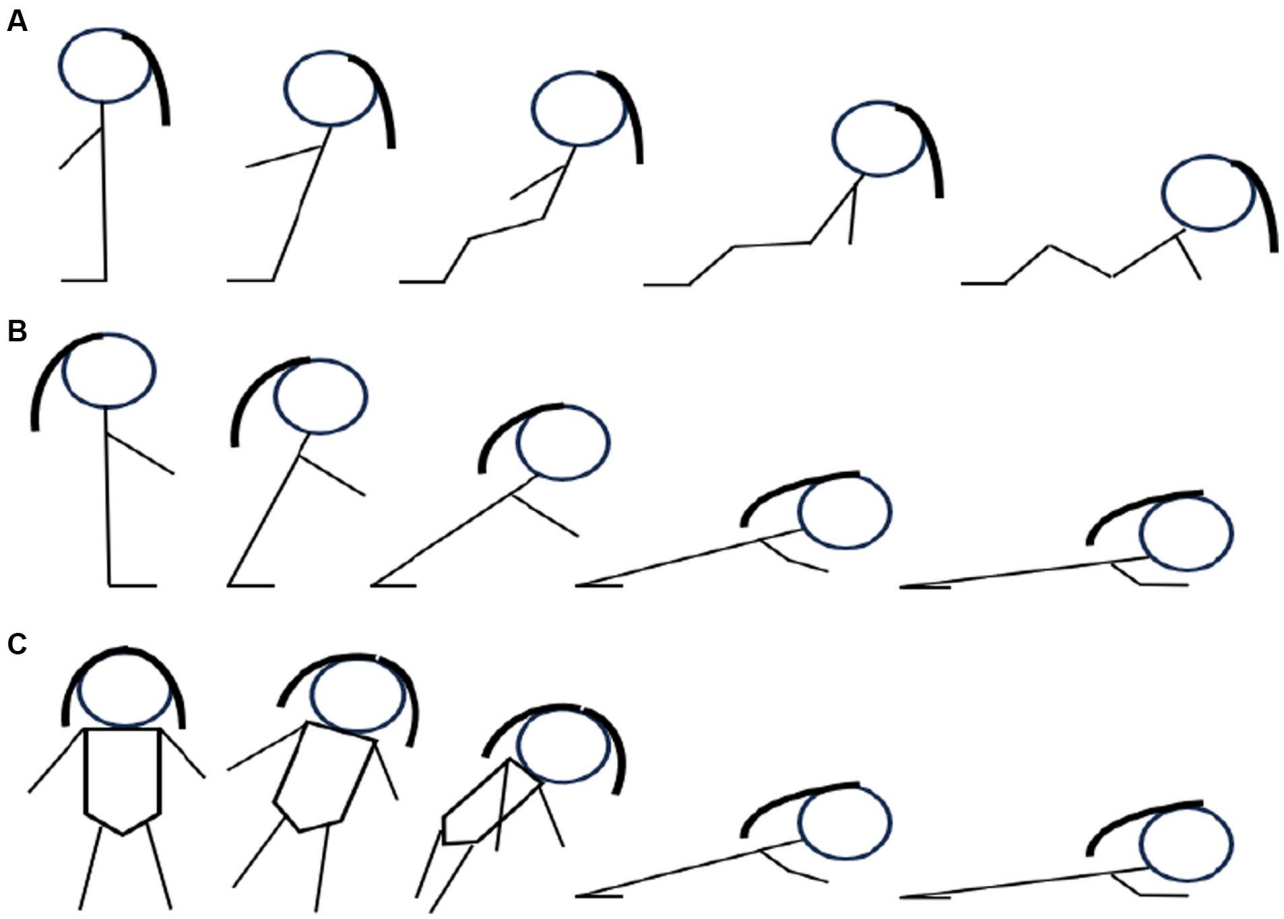
Safe landing techniques.

We have chosen the three safe landing techniques that have proven to be effective in reducing the impact severity of various falls (13) (Figure 2), namely:

- Backward squatting (flex the knees and hips while contracting the muscles spanning these joints);
- Forward elbow flexion (catch the ground with the outstretched arms while landing with the slightly flexed elbow);
- Side forward rotation (rotate forward during the descent to land on the outstretched hands).

To minimize the risk of injury, participants will practice the safe landing techniques on 12-inch thick gymnastics crash landing mats. The instructor will exemplify the technique before the participants. Each technique will be introduced gradually to protect the participants from falling directly at the beginning. In addition, two professionals will assist the participants’ movements to ensure their safety throughout the session. Participants will be encouraged to lower their center of gravity and increase the area of their body’s contact with the mats to decrease impact load. To help to protect the vital organs from damage, they must exhale to contract the muscles and constrict the rib cage on impact.

Participants will keep repeating the safe landing techniques to establish the conditioning reflex of instinctively and quickly assuming the movements to protect themselves, exha-le, relax, and allow the impact to spread through the body without injury.

To finalize, participants will perform five minutes of relaxation with active slow amplitude movements with music, stretching, and breathing exercises, followed by a brief group discussion to gather participant feedback on each exercise to guide future sessions.

The structure of the Safe landing training sessions is described in Table 2.

**Table 2:** Structure of the safe landing training sessions.

Phase 1: Warm-up (10 min)	Warm-up exercises to prepare participants for safe landing training. Mobility and flexibility exercises
Phase 2: Improvisation (10 min)	Participants have the freedom to move according to specific instructions. Encouraging participants to explore their movements and expand their physical capabilities.
Phase 3: Safe landing practice (20 min)	Guided practice of safe landing techniques with instructor supervision Participants practicing controlled landings and loss of balance. Participants practicing safe landing techniques independently. Instructor providing feedback and corrections as needed.
Phase 4: Cool-Down and Conclusion (5 min)	Cool-down exercises to relax muscles after safe landing practice. Summary of the key points covered in the safe landing training.

### 2.5. Data collection

Researchers defined the feasibility of trial design and procedures by six key domains for a subsequent registered randomized controlled trial. These key domains will be our primary outcomes and include:

- Recruitment strategy and rates (candidates screened, eligible, approached, consented, and excluded after screening).
- Enrollment (number of participants that attended the program).
- Retention (percentage of enrolled participants who completed final program assessments).
- Acceptability (assessed with an *ad hoc* survey and semi-structured exit interviews). The exit survey will consist of Likert scale questions to evaluate the participants’ satisfaction. The interviews aim to gain insight into participants’ perspectives on the intervention’s feasibility, acceptability, and usefulness and identify barriers and facilitators that may influence people with PD to participate in the program.
- Reasons for decline/withdrawal (the researchers will keep a record of reasons for decline/withdrawal).
- Adverse events (number of events involving injury).

A qualified research team member will collect secondary outcomes to identify an appropriate outcome and estimate parameters for a sample size calculation for a randomized controlled trial. These assessments will be performed in the week before (T0) and after completing the training program (T1) using the following instruments:

- FOF (Short FES-I). The Short FES-I is a self-report questionnaire used to assess an individual’s level of concern or FOF during various activities of daily living. The ratings are often done on a scale, typically ranging from 1 (not at all concerned) to 4 (extremely concerned). This scale had good test–retest reliability (*r* = 0.987) and good internal consistency (*α* = 0.958) (33);
- Clinical impairments (The Movement Disorder Society Unified Parkinson Disease Rating Scale - MDS-UPDRS) (44). The MDS-UPDRS is a comprehensive clinical assessment tool to evaluate non-motor and motor experiences of daily living and motor complications. It includes a motor evaluation and characterizes the extent and burden of disease. This scale had a test–retest reliability of 0.92 and internal consistency of 0.96 (45);
- Frequency and severity of falls (falls weekly registry). The registry collects data on falls, including when and where they occurred, the circumstances surrounding the falls, the individuals involved, and any resulting injuries (46);
- Gait (Timed Up Go - TUG). The Timed Up and Go Test is a reliable assessment tool for evaluating an individual’s mobility and functional balance, as it necessitates the ability to transition, walk, and change direction (46). Concerning the test–retest reliability of TUG, the intra-class correlation coefficient (95% CI) for the total score was equal to 0.96 and presented excellent internal consistency (*α* = 0.98) (47). Participants will begin in a seated position with their backs supported and are required to stand, walk 3 meters in a straight line, pivot, return to the chair, and sit down, mirroring the initial position. The test score is determined based on the participants’ time to complete the task. Completion within 10 s indicates normal mobility, while a duration of 11–20 s falls within the normal range for frail or partially independent adults with disabilities. A completion time exceeding 20 s is typically observed in individuals with a significant mobility impairment (46).
- Physical capacity (6 min walking distance test - 6MWD). The 6MWT evaluates a person’s exercise tolerance, functional capacity, and endurance. Participants are instructed to walk back and forth in a hallway as far as they can within 6 min. They can walk at their own pace, and they are allowed to slow down, stop, or rest during the test if needed (46). This test presented excellent test–retest reliability (intra-class correlation coefficient = 0.95–0.96) (48).

### 2.6. Data analysis

Quantitative method: the sample will be characterized using descriptive statistics, including measures such as count, mean, standard deviation, median, minimum, maximum, and range. Differences between the scores obtained from initial and final assessments for Short Falls Efficacy Scale-International (Short FES-I), Movement Disorder Society Unified Parkinson’s Disease Rating Scale (MDS-UPDRS), Timed Up Go (TUG), 6 Minutes Walking Distance (6MWD), and frequency and severity of falls will be evaluated using inferential statistics. Specifically, the parametric two-tailed paired samples t-test will be employed if the normality assumptions are met. Alternatively, the non-parametric two-tailed matched pairs Wilcoxon signed-rank test will be utilized in case of normality deviations. The statistical analysis will be conducted using the R statistical computing software.

Qualitative method: a research team member with a Ph.D. in Psychology will perform semi-structured interviews to obtain insight into participants’ perspectives on the intervention’s feasibility, acceptability, and usefulness and identify barriers and facilitators that may influence people with PD to participate in the program. This researcher is a skilled interviewer with no prior relationship with the participants. Two team members will transcribe verbatim the audio-recorded interviews into textual data using Microsoft Word. These researchers will independently perform an inductive thematic analysis process as described by Braun et al. (49). The analysis will be supported by QDA Miner Lite software. This process will allow the identification of themes emerging from the interview data through pre-analysis, encoding, categorization, and interpretation of the data.

### 2.7. Ethics and dissemination

Researchers will conduct this study following the Helsinki Declaration (as revised in 2013). The leading researcher is responsible for seeking approval from the institutional Ethics Committee and preserving the confidentiality of participants taking part in the study.

All participants will sign an informed consent form before any procedures. This form contains comprehensive information concerning the study aims, procedures, voluntariness, and possible risks of participation. Participants have the right to withdraw their consent to participate at any time without any consequence. However, all anonymized data collected may be applied in data analysis because this will not be linked to any identifiable participant information. All information will be kept strictly confidential. All information will be destroyed 5 years after the completion of the research project.

Researchers will submit the study results for publication in scientific journals and disseminate them at national and international PD conferences/seminars and PD communities.

## 3. Discussion

This research will assess the feasibility of the Landing Wise program, which combines group CBT with training of safe landing strategies. The program’s effectiveness will not be established in this study but will be the aim of a subsequent registered randomized controlled trial. The results of this study will decide whether it is feasible to proceed to a full trial and if any adjustments to procedures need to be made.

We expect that in addition to reducing the FOF, the program has the potential to challenge the balance system and be effective for balance outcomes and functional mobility.

Data from previous studies suggest that when applied separately, CBT and safe landing strategies are well accepted by the participants and have demonstrated efficacy (19, 20, 50, 51). However, it is essential to highlight that few studies employing safe landing strategies, including people with PD, were randomized controlled trials. It is also noteworthy that despite the positive health outcomes to the best of our knowledge, this is the first study that combines these interventions in people with PD.

The Landing Wise program can be an intervention adapted to the multidimensional impairments experienced by people with PD (e.g., motor, balance, and social impairments). CBT, a well-established psychotherapeutic approach, addresses cognitive processes, emotions, and behaviors. Individuals with PD are often challenged with physical motor impairments and cognitive and emotional challenges (24–26). The Landing Wise program integrates CBT to target these cognitive and emotional aspects, including FOF, negative thought patterns, and anxiety, which are prevalent in individuals with PD.

Furthermore, the program combines physical training with safe landing strategies to enhance motor skills and balance. Additionally, the group sessions within the Landing Wise program offer social interaction and support, potentially reducing social isolation and withdrawal for those with PD. However, further research is needed to establish the effects of our program on people with PD FOF, balance, and gait. Therefore, this study is the first step to determining the program’s feasibility for this population.

Although training safe landing techniques can be well accepted and enjoyable workout activity (50, 51), we acknowledge that it can also be intimidating for some people with FOF. However, the safety measures introduced by researchers can potentially mitigate this effect. In addition, the program has the potential to promote social interaction, as it will be performed in a group format. Thus, it is expected to develop a sense of camaraderie between participants (52, 53) and accomplishment (54), contributing to the intervention’s satisfaction (52, 54). Given all these aspects, combining CBT and safe landing strategies can be an especially well-adapted and attractive way of optimizing care for people with PD.

Like in previous research that employs a safe landing intervention, we expect the level of risks associated with being low (50, 51). However, the potential risks should be foreseen and addressed to ensure participants’ safety. Therefore, in addition to the protective gear and mattress, the instructor can adapt the exercise program to each participant’s skills and abilities to keep it challenging and engaging.

This study has limitations. First, as we intend to establish the program’s feasibility, the study procedures do not include a control group, which will prevent comparing the program’s effects to additional treatment. Second, when evaluating the program’s feasibility, we recognize the possibility of bias due to social desirability. As such, participants’ reports might diverge from their real perceptions and feelings. To minimize the effect of this bias during the qualitative assessment, we will implement practices recommended by Bergen and Labonté (55). Therefore, all study details will be explained clearly to participants, including confidentiality and anonymity procedures, how the data will be used, and the dissemination of results. We will conduct the interviews in a private location and not within earshot of others. The interviewer will resort to different methods to establish rapport with participants (e.g., humor, self-disclosure, making displays of respect).

If the interviewer suspects an answer indicates social desirability biases, he will maintain a nonconfrontational and respectful attitude and attempt to produce a more authentic reply by offering context when asking questions, recognizing that participants have different understandings, posing indirect questions, and requesting that participants provide examples to illustrate their answer.

## 4. Conclusion

This study aims to establish the feasibility of the Landing Wise program among people with PD. There is a growing interest in effective new forms of therapy in this population. However, the current literature has no studies on the use of combined group CBT with the training of safe landing strategies for people with PD. Therefore, this will be the first study to assess the program’s feasibility and preliminary effects. If proven, this study precedes the development of a randomized controlled trial that may prove the effectiveness of combined group CBT with the training of safe landing strategies as a therapeutic intervention for people with PD.

## Author contributions

JF, JD, CF, SF, IS, and CG: conceptualization. JF, JD, CF, CS, DS, FG, IC, JA, LM, SM, TS, SF, IS, NS, CR, and CG: methodology, writing-original draft preparation, writing-review, and editing. JF, and CG: supervision. JF: project administration. All authors have read and agreed to the published version of the manuscript.
